# Anterior cruciate ligament ruptures requiring surgical reconstruction are associated with decreased performance compared to matched controls in National Hockey League players

**DOI:** 10.1002/jeo2.70588

**Published:** 2026-03-22

**Authors:** Emmitt Hayes, Pamela Garcia Pulido, Sasha Carsen, Bogdan Matache, Michael Pickell

**Affiliations:** ^1^ Division of Orthopaedic Surgery The University of Ottawa Ottawa Ontario Canada; ^2^ Division of Orthopaedic Surgery Children's Hospital of Eastern Ontario Ottawa Ontario Canada

**Keywords:** analytics, athletic performance outcomes, knee injury, return to sport, surgical outcomes

## Abstract

**Purpose:**

To evaluate National Hockey League (NHL) player productivity and effectiveness following anterior cruciate ligament (ACL) reconstruction using advanced hockey statistics.

**Methods:**

NHL players who underwent ACL reconstruction between 2008 and 2022 were identified using the NHL Injury Viz database. Players with multiligament injuries, reruptures, players who had complications requiring revision surgery within the study period, players without pre‐ or postinjury performance data, goaltenders and players who sustained another orthopaedic injury requiring surgical management during the study period were excluded. Demographic and outcome data for 1‐year preinjury and 2‐year postinjury were abstracted. The primary outcome was wins above replacement per 60 min played (WAR/60). Secondary outcomes were offensive and defensive goals above replacement per 60 (GAR/60) min played. Controls were NHL players matched by position, draft year and index season performance. Pre‐ and postinjury outcomes were compared with paired *t*‐tests for normally distributed variables and the Wilcoxon signed rank test for nonnormally distributed variables.

**Results:**

Sixty‐one ACL ruptures were identified, of which 43 were eligible for inclusion. Preinjury, postinjury year 1 and postinjury year 2 WAR/60 were 0.05, 0.02 and 0.03 in cases (*p* = 0.01, *p* = 0.03 compared to preinjury) and 0.04, 0.05 and 0.08 in controls (*p* = 0.50, *p* = 0.00, *p* = 0.00 compared to cases). Preinjury, postinjury year 1 and postinjury year 2 offensive GAR/60 were 0.24, 0.13 and 0.18 in cases (*p* = 0.04, *p* = 0.03 compared to preinjury) and 0.16, 0.27 and 0.37 in controls (*p* = 0.83, *p* = 0.02, *p* = 0.01 compared to controls). Preinjury, postinjury year 1 and postinjury year 2 defensive GAR/60 were 0.04, −0.01 and 0.02 in cases (*p* = 0.17, *p* = 0.39 compared to preinjury) and −0.03, 0.01 and 0.03 in controls (*p* = 0.08, *p* = 0.60, *p* = 0.89 compared to controls).

**Conclusions:**

ACL ruptures requiring surgical reconstruction are associated with significantly decreased performance in NHL players compared to preinjury and matched controls, driven by significantly decreased offensive performance.

**Level of Evidence:**

Level III, therapeutic case‐control study.

## Graphical abstract

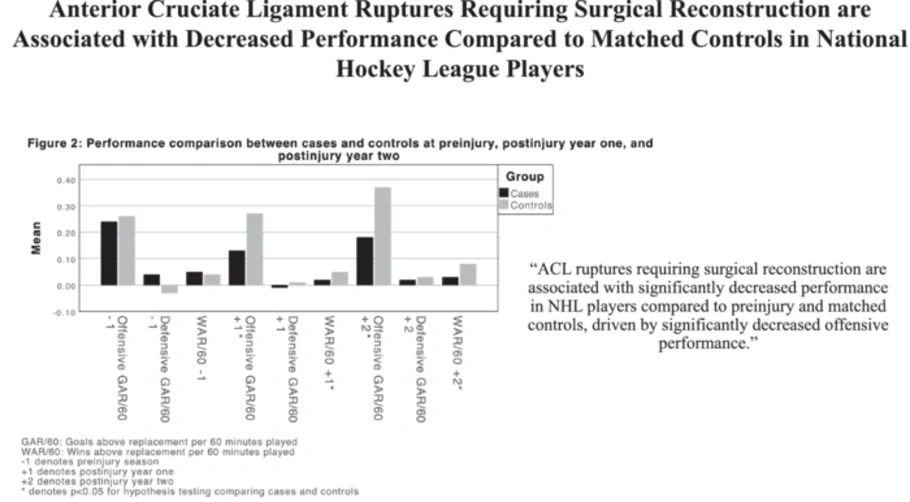

**Abbreviations**
ACL: anterior cruciate ligament
GAR/60: goals above replacement per 60 min played
NHL: National Hockey League
RAPM: regularised adjusted plus minus
WAR/60: wins above replacement per 60 min played

## INTRODUCTION

Anterior cruciate ligament (ACL) ruptures occur at a rate of 0.42 per 1000 game hours in National Hockey League (NHL) players [8] and are associated with high rates of return to sport (95%) [8] but shortened career duration compared to healthy controls [17]. Prior research has found that players returning from ACL reconstruction sustain a decrease in games played (GP), goals, assists and points [8, 17]. Prior research evaluating outcomes following ACL reconstruction in NHL players has relied on traditional hockey statistics including metrics such as goals scored, assists, points and shots on goal [5, 8, 17]. While these statistics are widely understood and provide a general evaluation of a player's individual performance, they are based on low frequency events which causes them to be influenced by randomness and limits their ability to predict future outcomes [1, 13]. Furthermore, traditional statistics do not account for teammate/opponent performance or coaching decisions including ice time and situational usage and thus provide an imperfect assessment of an individual player's performance [10]. As such, there is a chance that prior studies have either over or underestimated performance changes following ACL reconstruction in NHL players, necessitating further study.

Advanced statistics address these limitations by relying on high occurrence events (shots or scoring chances) to minimise the effect of randomness, incorporate statistical adjustments for teammate/opponent performance and game situation, and express outcomes per unit time to account for differences in player usage. Collectively, advanced statistics may allow for a more comprehensive analysis of individual level performance changes following ACL reconstruction than prior work.

Understanding the impact of ACL injury on sports performance is important for setting treatment expectations, predicting performance and comparing treatments. The purpose of this study was to assess NHL player productivity and effectiveness following ACL reconstruction using advanced hockey statistics including offensive and defensive goals above replacement and wins above replacement. Based on prior work demonstrating performance declines following ACL reconstruction in NHL players using traditional statistics [8, 17], the hypothesis of this study was that there will be declines in overall, offensive, and defensive effectiveness when athletes return to play following ACL reconstruction.

## METHODS

Institutional review board approval was not required for this study as publicly available data was used. A retrospective case‐control study was performed by identifying NHL players who underwent ACL reconstruction between the 2008/2009 and 2021/2022 seasons. Like prior studies evaluating sports performance following injury, cases were identified in a systematic fashion using publicly available data [5, 6, 8, 9, 11, 15].

NHL players who sustained a knee injury during the study period were identified using the publicly available NHL Injury Viz database of knee injuries, which tracks every game missed and the type of injury causing the missed game in NHL players [14]. The NHL Injury Viz database has been used extensively in prior work evaluating outcomes following orthopaedic injuries in NHL players [7, 18]. The NHL Injury Viz database was queried 15 October 2023. News articles, press releases, injury reports, player profiles and biographies were reviewed between 15 October and 20 November 2023 for each player identified via database searching to determine whether each player sustained an ACL rupture and underwent surgical reconstruction as well as the date of injury and date of surgery. The database was searched in the same fashion for all included players to determine whether they had other orthopaedic injuries requiring surgical management during the study period.

Players who sustained a native ACL rupture and underwent surgical ACL reconstruction during the study period, and who had played at least one NHL season prior to injury were included. Players who did not undergo ACL reconstruction, players with chronic ACL ruptures (one player who had sustained an ACL rupture prior to the study period and played multiple seasons before undergoing surgical reconstruction), players undergoing multi ligament knee reconstruction, players who sustained a repeat rupture of a previously reconstructed ACL, players who sustained a repeat rupture or contralateral ACL rupture within two subsequent seasons, players who had complications requiring revision surgery within the first 2 years following surgery, players who did not have a full season of pre or postinjury performance data, goaltenders and players who sustained another orthopaedic injury requiring surgical management within the study period were excluded. Players who returned to play only a single postinjury season were included and analysed at postinjury year 1 year but excluded from analysis at postinjury year 2.

Birth date, position, draft year, draft position, handedness, GP, height and weight were obtained from Hockey Reference (Hockeyreference.com). Outcome data were obtained from Evolving‐Hockey (https://evolving-hockey.com/), a website providing access to traditional and advanced hockey statistics that has previously been used in sports science research [2]. Evolving Hockey uses NHL Real Time Scoring System data that has been collected by the NHL since 2007. During all games, the NHL records the score, situation (even strength, power play and penalty kill), and on ice location during certain events (giveaways, takeaways, hits, faceoffs, shots, blocked shots and penalties) [19]. These data are made available for statistical analysis. Definitions and descriptions of outcome measures are available in Table 1. The primary outcome was wins above replacement per 60 min played (WAR/60). Wins above replacement is a statistic that quantifies the value a player adds to their team per 60 min of ice time measured in terms of wins relative to a replacement level player, where a replacement level player is defined as all forwards below the top 13 forwards and defencemen below the top 7 defenceman in time on ice per team per season [19, 20, 21]. A value greater than zero indicates that a player adds value above a replacement level player, while a negative number indicates that a player provides less value than a replacement level player. This statistic is adjusted for teammate and opponent performance to provide an assessment of an individual player's value that is not impacted by the quality of their team, difficulty of their schedule, and whether a team makes the playoffs. Wins Above Replacement per 60 min played was chosen as the primary outcome measure as it summarises a player's overall value to their team in a single metric, encompassing offensive, defensive and special teams' performance while adjusting for teammate and opponent skill level.

**Table 1:** Description of advanced analytic metrics in National Hockey League (NHL) players.

Metric	Definition
Expected Goal	The probability of a scoring chance resulting in a goal based on the shot location. Expected goals for an individual player refers to the sum of the expected goals value of all scoring chances generated by a player per unit time.
Corsi (%)	The percentage of shot attempts made by a player's team compared to the opposing team when a player is on the ice (Shot attempts by player's team/[Shot attempts by player's team + shot attempts by opponent]). >50% indicates that a team is generating more short attempts than their opponent when a given player is on the ice.
Expected goals (%)	Expected goals scored by a player's team divided by expected goals scored by the opposing team while a player is on the ice. >50% indicates that a team is generating more expected goals than their opponent when a given player is on the ice.
Regularised adjusted plus minus (RAPM) expected goal differential per 60 min played	Expected goals scored by a player's team divided by expected goals scored by the opposing team while a player is on the ice adjusted by regularised linear regression for the effects of teammate performance, opponent performance, the score of the game, the effect of playing at home versus away, the effects of playing back to back games and the effect of being on the ice for a shift that started in the offensive or defensive zone [22].
RAPM Corsi differential per 60 min played	Corsi % while a player is on the ice adjusted by regularised linear regression for the effects of teammate performance, opponent performance, the score of the game, the effect of playing at home versus away, the effects of playing back to back games and the effect of being on the ice for a shift that started in the offensive or defensive zone [22].
RAPM expected goals for per 60 min played	Expected goals scored by the player's team while the player is on the ice adjusted by regularised linear regression for the effects of teammate performance, opponent performance, the score of the game, the effect of playing at home versus away, the effects of playing back to back games and the effect of being on the ice for a shift that started in the offensive or defensive zone [22].
RAPM expected goals against per 60 min played	Expected goals scored against the player's team while the player is on the ice adjusted by regularised linear regression for the effects of teammate performance, opponent performance, the score of the game, the effect of playing at home versus away, the effects of playing back to back games, and the effect of being on the ice for a shift that started in the offensive or defensive zone [22].
RAPM Corsi for per 60 min played	Shot attempts by a player's team while the player is on the ice adjusted by regularised linear regression for the effects of teammate performance, opponent performance, the score of the game, the effect of playing at home versus away, the effects of playing back to back games, and the effect of being on the ice for a shift that started in the offensive or defensive zone [22].
RAPM Corsi against per 60 min played	Shot attempts against a player's team while the player is on the ice adjusted by regularised linear regression for the effects of teammate performance, opponent performance, the score of the game, the effect of playing at home versus away, the effects of playing back to back games, and the effect of being on the ice for a shift that started in the offensive or defensive zone [22].
Expected offensive goals above replacement per 60 min played	RAPM expected goals for combined with even strength and power play zone starts, hits, giveaways, takeaways, faceoff percent and shots in a multivariate regression model to give an overall estimate of offensive performance expressed as the number of offensive goals added (or subtracted) relative to a replacement level player per 60 min played, where a replacement level player is defined as all forwards below the top 13 forwards and defencemen below the top 7 defenceman in time on ice per team per season [19, 20, 21].
Expected defensive goals above replacement per 60 min played	RAPM expected goals against combined with even strength and power play zone starts, hits, giveaways, takeaways and faceoff percent in a multivariate regression model to give an overall estimate of defensive performance expressed as the number of offensive goals added (or subtracted) relative to a replacement level player per 60 min played, where a replacement level player is defined as all forwards below the top 13 forwards and defencemen below the top 7 defenceman in time on ice per team per season [19, 20, 21].
Expected wins above replacement per 60 min played (WAR/60)	The sum of expected offensive and defensive goals above replacement for an individual player converted from goals to wins to give an estimate of overall performance expressed as the number of wins added (or subtracted) relative to a replacement level player per 60 min played, where a replacement level player is defined as all forwards below the top 13 forwards and defencemen below the top 7 defencemen in time on ice per team per season [19, 20, 21].

Secondary outcomes included GP, total expected goals generated per 60 min played, Corsi percentage, expected goals percentage, regularised adjusted plus minus (RAPM) expected goal percentage, RAPM Corsi percentage, RAPM expected goals for/against, RAPM Corsi for/against, expected offensive goals above replacement per 60 min played, and expected defensive goals above replacement per 60 min played. A detailed description of the methodology behind each statistic is available on the Evolving‐Hockey website and in Table 1 [19, 20, 21, 22]. Data were collected from the first full season prior to injury and for the following two seasons after injury.

### Cohort

A 1:1 matched cohort of players without a history of ACL injury was created. For each case, a matched player of the same draft year, position, within 2 years of age, active during the study period, and with similar index wins above replacement per 60 min played was selected for inclusion in the cohort.

### Statistical analysis

Data analysis was performed using IBM SPSS Statistics version 29. Outcome data were assessed for normality using the Shapiro–Wilk test. Hypothesis testing was performed using paired *t*‐tests for normally distributed variables and with the Wilcoxon–Signed rank test for nonnormally distributed variables. A *p*‐value of <0.05 was used as a cut‐off for a statistically significant result.

### Power calculation

All eligible players who sustained an ACL rupture beginning one season after the NHL began collecting advanced player data in the 2007/2008 season were included. All eligible players who underwent ACL reconstruction 1 year after the NHL began collecting the shot data necessary for calculating goals and wins above replacement were included. An ex‐ante power calculation was performed. Using the mean preinjury WAR/60 in controls of 0.045 with a standard deviation of 0.068 and assuming 15 min of ice time per game over an 82 game season, 31 subjects per group would be required to ascertain a performance difference of 1 win above replacement per season with alpha of 0.05 and power of 0.8.

## RESULTS

Seven hundred and eleven knee injury reports in 446 players from the 2008/2009 to the 2021/2022 seasons were reviewed and 61 individual players who had sustained an ACL rupture within this time period were identified. Seven players who had an injury requiring a separate orthopaedic procedure during the study period, three players who sustained a retear within the study period, three players without a full season of preinjury data, one player who tore the contralateral ACL during the study period, one player with a chronic ACL tear, one player who underwent multiligamentous knee reconstruction, one player who had reoperation for infection within the study period, one player who did not undergo surgery and retired, one goaltender and four players who failed to return were excluded. Three additional players were identified through other news sources and two players sustained contralateral tears outside of the study period and so both knees were included, leaving 43 knees in 41 players for inclusion. Players returned to play a mean of 300 (standard deviation 102) days from injury. Figure 1 displays a flow diagram of patients through this study. Demographic characteristics are presented in Table 2. Of note, there were no significant differences between cases and controls in age, height, weight, BMI or draft position.

**Figure 1:**
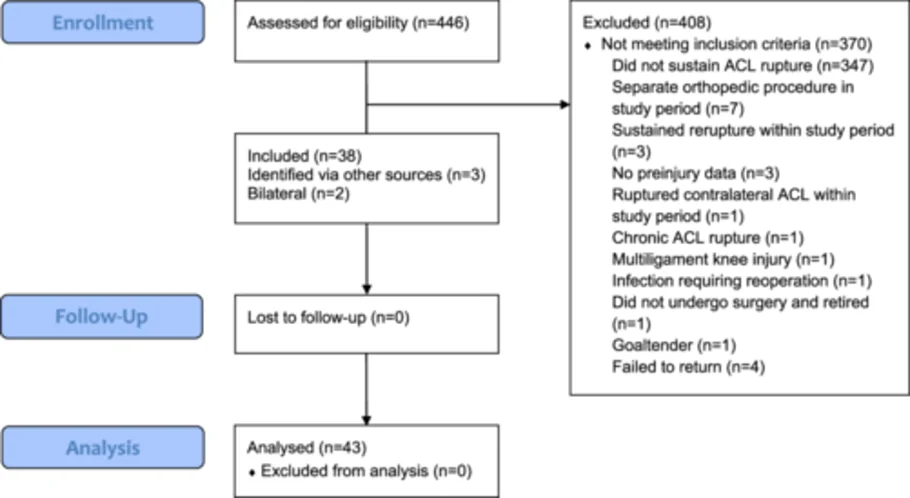
CONSORT diagram demonstrating the flow of patients through this study.

**Table 2:** Demographic data among cases and controls reported as mean (standard deviation).

	Cases	Controls	*p*
*n*	43	43	
Age (years) at injury	27.7 (4.1)	28.0 (4.2)	0.30
Height (cm)	185.7 (3.6)	186.7 (5.2)	0.20
Weight (kg)	91.7 (5.6)	93.1 (8.3)	0.34
Body mass index (kg/m^2^)	26.7 (1.3)	26.7 (1.6)	0.90
Draft pick number	74.1 (73.0)	55.1 (62.2)	0.79

Table 3 displays outcome data for the index, postinjury year 1 and postinjury year 2 seasons. Of note, expected wins above replacement per 60 min played were significantly decreased compared to preinjury at both postinjury year 1 and postinjury year 2 (*p* = 0.01, *p* = 0.03, respectively) due to significantly decreased offensive goals above replacement per 60 min played both 1‐ and 2‐years following surgery (*p* = 0.04, *p* = 0.03, respectively).

**Table 3:** Comparison of preinjury and postinjury performances among cases at baseline (Most recent full season prior to anterior cruciate ligament [ACL] rupture/reconstruction), 1 year postsurgery and 2 years postsurgery.

Preoperative and postoperative performance‐cases					
	Injury −1	Injury +1	*p*	Injury +2	*p*
*n*	43	43			
Games played	54.9	52.3	0.42	55.1	0.16
Expected goals per 60 min played	0.54	0.60	0.53	0.57	0.30
Corsi percentage	49.7	48.5	0.09	49.2	0.28
Expected goals percentage	50.1	50.0	0.52	49.2	0.20
RAPM expected goal percentage per 60 min played	0.03	−0.01	0.15	0.02	0.29
RAPM Corsi percentage per 60 min played	0.86	−0.66	0.01	−0.04	0.10
RAPM expected goals for per 60 min played	0.02	−0.01	0.18	0.00	0.21
RAPM expected goals against per 60 min played	−0.01	0.00	0.71	−0.02	0.94
RAPM Corsi for per 60 min played	0.37	−0.30	0.07	0.09	0.24
RAPM Corsi against per 60 min played	−0.50	0.36	0.03	0.13	0.15
Expected offensive goals above replacement per 60 min played	0.24	0.13	0.04	0.18	0.03
Expected defensive goals above replacement per 60 min played	0.04	−0.01	0.17	0.02	0.39
Expected wins above replacement per 60 min played	0.05	0.02	0.01	0.03	0.03

*Note*: *p* values for comparison of performance at 1 and 2 years postsurgery with presurgery.

Abbreviation: RAPM, regularised adjusted plus minus.

Table 4 and Figure 2 display a comparison of cases and controls at index, postinjury year 1 and postinjury year 2. Notably, there was no significant difference in GP, offensive, or defensive performance in the index season (*p* = 0.07, *p* = 0.83, *p* = 0.08, respectively). GP at postinjury year one were significantly decreased compared to controls (*p* = 0.00). Offensive expected goals above replacement and expected wins above replacement were significantly decreased compared to controls at all postinjury time points (*p* = 0.02, *p* = 0.01, *p* = 0.00, *p* = 0.00 for offensive expected goals above replacement and expected wins above replacement at postinjury year 1 and year 2, respectively). There was no significant postinjury difference in defensive performance between cases and controls (*p* = 0.60, *p* = 0.89 at postinjury year 1 and year 2, respectively).

**Table 4:** Comparison of pre‐ and postinjury performance between cases and matched controls at baseline (most recent full season prior to ACL rupture/reconstruction), 1‐year postsurgery and 2‐year postsurgery.

	Injury −1 cases	Injury −1 controls	*p*	Injury +1 cases	Injury +1 controls	*p*	Injury +2 cases	Injury +2 controls	*p*
Games played	54.9	61.5	0.07	52.3	67.2	0.00	55.1	63.0	0.23
Expected offensive goals above replacement per 60 min played	0.24	0.26	0.83	0.13	0.27	0.02	0.18	0.37	0.01
Expected defensive goals above replacement per 60 min played	0.04	−0.03	0.08	−0.01	0.01	0.60	0.02	0.03	0.89
Expected wins above replacement per 60 min played	0.05	0.04	0.50	0.02	0.05	0.00	0.03	0.08	0.00

**Figure 2:**
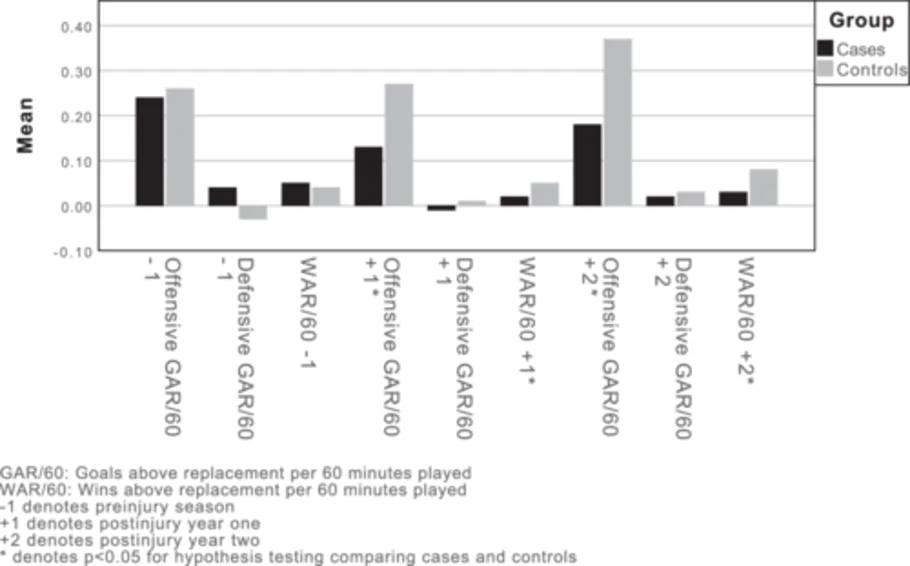
Performance comparison between cases and controls at preinjury, postinjury year 1 and postinjury year 2.

## DISCUSSION

The primary finding of this study was that ACL ruptures requiring surgical reconstruction are associated with significantly decreased hockey performance as measured by wins above replacement per 60 min played compared to preinjury and matched controls. This result is driven by significantly decreased offensive performance measured by offensive goals above replacement per 60 min played compared to preinjury and matched controls. These results suggest that ice hockey performance is decreased in NHL players who sustain an ACL rupture requiring surgical reconstruction. Overall wins above replacement remain positive at 1 and 2 years postinjury, suggesting that on average players are still performing at a level above a replacement level player following injury.

Prior research has demonstrated that ACL ruptures are associated with decreased career duration [17], GP [17], goals [8, 17], assists [17], points [8, 17] and performance score [11] in NHL players. Other studies have found no difference in career duration [5, 8] or GP [8] and one study found that cases scored more goals and points than controls [5]. Compared to prior studies that assessed performance using traditional statistics, this study found a significant overall performance decline at 1 and 2 years postinjury driven by decreased offensive performance with no change in defensive performance. While prior literature does give a general picture of the effect of ACL rupture on postinjury performance, the statistics used in these studies are influenced by randomness [1] (a possible reason for conflicting prior results) and are vulnerable to influence from strategy, usage, teammate performance, game script and other factors not directly reflective of a player's individual profile. Traditional statistics have been shown to be relatively poor predictors of team performance compared to advanced statistics, suggesting that they may fail to capture as much information as advanced statistics [1, 13]. It is the authors' opinion that the advanced statistics used in this study better characterise the performance of an individual player than metrics used in prior studies and thus lead to more generalisable results than prior work.

Advanced statistics have been used in research evaluating outcomes following orthopaedic injuries and procedures in other sports. For example, player efficiency rating has been used to evaluate performance changes following ACLR in professional basketball players [3], while wins above replacement has been used in baseball to describe performance changes following ulnar collateral ligament reconstruction (UCLR) and to compare outcomes with different graft choices in UCLR [4, 12, 16]. Given the previously described limitations of traditional statistics, advanced statistics may offer a more accurate and nuanced view of performance than traditional statistics.

The primary finding in this study of a decrease in performance at all time points following ACL reconstruction suggests that while players may return to play at high rates following ACL reconstruction, the mean player who returns to play does not return to their preinjury level of performance or the expected performance level of a matched control player. This finding maybe helpful for counselling elite ice hockey players following ACL reconstruction, though further research is needed to determine whether similar performance changes are found in junior or subelite hockey players.

### Limitations

This study has several limitations. The use of publicly available data induces possible observer bias as there is the possibility that some players were missed that had differing performance outcomes compared to included players. However, publicly available data is frequently used in studies comparing outcomes following orthopaedic surgical procedures [5, 8, 11] and was improved upon in this study by using a publicly available database previously compiled in a systematic fashion. The use of publicly available data induces possible selection bias as more well‐known players may have had more readily available injury and surgical data. Furthermore, the publicly available data used did not contain information on individual injuries, concomitant injuries (such as meniscal or cartilage injury), other risk factors, surgical technique, concomitant procedures, or rehabilitation protocols used, precluding further stratification and analysis. Finally, while all eligible players were included, the sample was limited by the small number of NHL players who sustained an ACL rupture during the study period, which may limit the statistical power of this analysis and the generalisability of the results.

## CONCLUSION

ACL ruptures are associated with significantly decreased hockey performance compared to preinjury and matched controls in professional athletes. This is driven by decreased offensive performance when compared to preinjury and matched controls. These results suggest that hockey performance may fail to return to baseline following ACL reconstruction in ice hockey players. Further research is needed to determine the relationship between ACL rupture and subsequent performance in youth and amateur hockey players.

## AUTHOR CONTRIBUTIONS

**Emmitt Hayes**: Study design, data collection, data analysis/interpretation, manuscript writing, final approval. **Pamela Garcia Pulido**: Study design, data analysis/interpretation, manuscript writing, final approval. **Sasha Carsen**: Study design, data analysis/interpretation, manuscript writing, final approval. **Bogdan Matache**: Study design, data analysis/interpretation, manuscript writing, final approval. **Michael Pickell**: Study design, data analysis/interpretation, manuscript writing, final approval.

## CONFLICT OF INTEREST STATEMENT

Michael Pickell: Consultant for ConMed and Arthrex. Bogdan Matache: Has received research support from Arthrex and Pendopharm for unrelated previous studies. Sasha Carsen: Has performed educational consulting for Stryker and Smith and Nephew. The remaining authors declare no conflicts of interest.

## ETHICS STATEMENT

Institutional review board approval was not required for this study as publicly available data were used.

## Data Availability

The data sets analysed in this study are publicly available and can be accessed from publicly accessible sources. No new data were generated during this study. Analysed raw data from the current study are available from the corresponding author on reasonable request.
